# Gametocytes from K13 Propeller Mutant Plasmodium falciparum Clinical Isolates Demonstrate Reduced Susceptibility to Dihydroartemisinin in the Male Gamete Exflagellation Inhibition Assay

**DOI:** 10.1128/AAC.01426-18

**Published:** 2018-11-26

**Authors:** Sonia Lozano, Pablo Gamallo, Carolina González-Cortés, Jesús-Luís Presa Matilla, Rick M. Fairhurst, Esperanza Herreros, Chanaki Amaratunga, Janneth Rodrigues

**Affiliations:** a Diseases of the Developing World (DDW), Global Health R&D, GlaxoSmithKline, Tres Cantos, Madrid, Spain; b Laboratory of Malaria and Vector Research, National Institute of Allergy and Infectious Diseases, National Institutes of Health, Rockville, Maryland, USA

**Keywords:** DHA, antimalarial agents, artemisinin, exflagellation, gametocytes

## Abstract

Mutations in the kelch propeller domain (K13 propeller) of Plasmodium falciparum parasites from Southeast Asia are associated with reduced susceptibility to artemisinin. We exposed *in vitro*-cultured stage V gametocytes from Cambodian K13 propeller mutant parasites to dihydroartemisinin and evaluated the inhibition of male gamete formation in an *in vitro* exflagellation inhibition assay (EIA). Gametocytes with the R539T and C580Y K13 propeller alleles were less susceptible to dihydroartemisinin and had significantly higher 50% inhibitory concentrations (IC_50_s) than did gametocytes with wild-type alleles.

## Abstract (precis)

Mutations in the kelch propeller domain (K13 propeller) of Plasmodium falciparum parasites from Southeast Asia are associated with reduced susceptibility to artemisinin. We exposed *in vitro*-cultured stage V gametocytes from Cambodian K13 propeller mutant parasites to dihydroartemisinin and evaluated the inhibition of male gamete formation in an *in vitro* exflagellation inhibition assay (EIA).

## TEXT

Artemisinin-based combination therapies (ACTs) are recommended as a first-line treatment for uncomplicated Plasmodium falciparum malaria (1). ACTs comprise a fast-acting artemisinin derivative and a long-acting partner drug (e.g., lumefantrine, mefloquine, and piperaquine) and are key contributors to the recent successes of global malaria control (1). However, reduced susceptibility to artemisinin (as measured by a slow parasite clearance rate) and clinical failure of ACTs (recrudescences) have recently been reported from the Greater Mekong Subregion (2–5). A novel *in vitro* ring-stage survival assay (RSA), in which 0- to 3-h ring-stage parasites from culture-adapted isolates from Cambodian patients are exposed to a pharmacologically relevant dose of dihydroartemisinin (DHA) (700 nM for 6 h), showed increased survival in parasites from slow-clearing versus fast-clearing infections (6). Reduced susceptibility to artemisinin was associated with single nucleotide polymorphisms in the propeller domain of a P. falciparum kelch protein (K13 propeller) (7). Cambodian isolates with the C580Y, Y493H, and R539T K13 propeller mutations were associated with slow parasite clearance rates and higher RSA survival rates (7–9).

Mature sexual-stage parasites (gametocytes) in peripheral blood are necessary for transmission of Plasmodium spp. to mosquitoes. Artemisinins are effective against immature gametocytes (10, 11) but show reduced efficacy against mature gametocytes (12), which can persist for weeks after treatment and thus contribute to onward transmission (13, 14). The ability of gametocytes from K13 propeller mutant parasites to withstand artemisinin exposure and remain functionally viable in patients following ACT could additionally impact transmission dynamics in the Greater Mekong Subregion. Few studies have characterized gametocytes from fast- and slow-clearing Cambodian isolates. Ashley et al. (4) reported a higher incidence of gametocytemia in patients with slow-clearing K13 propeller mutant parasites. St. Laurent et al. (15) showed that *in vitro*-cultured gametocytes from these mutant parasites could infect highly diverse Anopheles species from Southeast Asia and Africa.

We sought to explore the effect of DHA on *in vitro*-cultured mature stage V gametocytes from K13 propeller mutant and wild-type (WT) Cambodian clinical isolates in an exflagellation inhibition assay (EIA). The EIA is based on the ability of functionally viable mature male gametocytes to undergo a time- and temperature-dependent release of exflagellating male gametes, and it measures the ability of a compound to inhibit the number of exflagellation centers per microscopic field in drug-treated gametocyte cultures compared to dimethyl sulfoxide (DMSO)-treated controls. We recently demonstrated the ability of this assay to predict the transmission-blocking behavior of antimalarials in mosquitoes infected with P. falciparum in the standard membrane feeding assay (16). In the present study, we compared the DHA susceptibilities of mature gametocytes from slow- and fast-clearing Cambodian isolates (17) (Table 1) by estimating their 50% inhibitory concentration (IC_50_) in EIA. (All methods in this study were carried out in accordance with GlaxoSmithKline guidelines and regulations. Human red blood cells were obtained from Biobank of Castilla y León, BST, and the Centro de Transfusiones de Madrid, and the relevant ethics committee approvals were obtained from Autonomous University of Madrid [CEI-45-890] to enable the use of these samples from blood donors. All use of human biological samples in this study was in accord with the terms of the informed consents given by the sample donors.)

**TABLE 1:** Cambodian *P. falciparum* clinical isolates used in this study

Isolate	Parasite clearance half-life (h)^*a*^	K13 propeller allele^*b*^
959	4.96	WT
937	5.58	WT
845	8.05	C580Y
968	7.97	C580Y
957	6.87	R539T
851	6.72	R539T

a Parasite clearance half-life was calculated based on posttreatment 6-hourly parasite density values, using the parasite clearance estimator developed by the Worldwide Antimalarial Resistance Network (17).

b K13 propeller allele was determined from whole-genome sequence data (8).

Gametocyte cultures were generated as described previously (16). Five-milliliter cultures containing 1% to 3% stage V gametocytes were incubated with increasing concentrations of DHA for 6 h (short exposure) or 48 h (long exposure). Controls comprising gametocytes exposed to the same final concentration of DMSO (0.1%) were processed in parallel. After the 6-h incubation, short-exposure cultures were centrifuged at 500 × *g* for 5 min, and the supernatant with the remaining DHA was removed by aspiration, replenished with fresh medium, and further incubated for a total duration of 48 h. Percent exflagellation inhibition (EI) was determined at 6 and 48 h. DHA was not removed for long-exposure treatments, and the EI was measured at 48 h. For EI determination, 100 µl of DHA-exposed and DMSO-exposed cultures were centrifuged at 500 × *g* for 30 s, and the packed cells were resuspended in 15 µl of prewarmed (37°C) medium, introduced into a hemocytometer, and incubated at room temperature (16). Movement of exflagellation centers was recorded between minutes 18 and 22 by video microscopy and quantified using a semiautomated method, and the EI was calculated for each treatment as previously described (16, 18), using the equation EI = [(*E_C_* − *E_T_*)/*E_C_*] × 100, where *E_C_* and *E_T_* are the number of exflagellation centers per field in the control and the DHA-treated sample, respectively. EI versus DHA concentration was fitted to a normalized sigmoidal dose-response curve (19) with two parameters (log 50% inhibitory concentration [logIC_50_] and Hill slope). The *F* test performed on the sum of squares to compare fits and implemented in GraphPad Prism version 6.07 (19) was used to compute statistical significance, comparing the estimated (best-fit) logIC_50_ parameter for isolates with the R539T and C580Y versus WT alleles.

**FIG 1:**
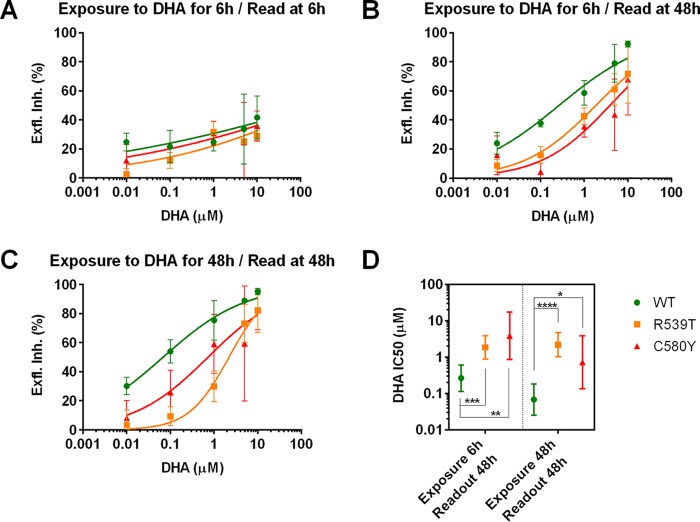
Dose response of exflagellation inhibition (Exfl. Inh.) (%) relative to control versus DHA concentration grouped by K13 propeller allele (WT in green, R539T in orange, and C580Y in red) for short exposure (6 h) and read at 6 h (A) or 48 h (B) and for long exposure (48 h) read at 48 h (C). The points represent the mean of the measured values, the error bars represent the standard error of the means, and the colored lines represent the corresponding best fit curves (normalized sigmoidal function with two parameters). (D) EI IC_50_ of WT, R539T, and C580Y isolates for different exposures. The bars represent the 95% confidence interval for each estimate. For each treatment, the numbers of independent replicates per isolate were as follows: isolate 959, 3; isolate 937, 2; isolate 845, 2; isolate 968, 2; isolate 957, 2; and isolate 851, 7. *, *P* ≤ 0.05; **, *P* < 0.01; ***, *P* < 0.001; ****, *P* < 0.0001.

With two isolates per allelic group, the DHA IC_50_ for gametocytes from K13 propeller mutant isolates was significantly higher than that for the WT isolates (Fig. 1). Inhibition of male gamete formation by short exposure of gametocytes to DHA did not permit EI IC_50_ estimation at 6 h in the dose range used (Fig. 1A). However, when the same 6-h DHA-exposed gametocytes were subjected to EIA at 48 h, the IC_50_ could be estimated (Fig. 1B), suggesting that short exposure to DHA results in a delayed impairment of their ability to form male gametes. The IC_50_s of R539T and C580Y gametocytes were significantly higher than that of the WT, showing that gametocytes from K13 propeller mutant slow-clearing isolates were better able to tolerate DHA exposure than were the WT fast-clearing isolates (Fig. 1D). Given the short plasma half-life of DHA, the short exposure time of 6 h mimics *in vivo* conditions, as it does in the RSA. We explored the effect of a long exposure time of 48 h in the EIA (Fig. 1C), which again showed significantly higher IC_50_s for the K13 propeller mutants than for the WT gametocytes. These results indicate that measuring the EI at 48 h in both short and long exposures can differentiate between gametocyte susceptibility profiles to DHA. Long exposure to DHA resulted in ∼4-fold and 5-fold reductions in the IC_50_s of WT (0.27 and 0.068 µM, respectively) and C580Y (3.9 and 0.73 µM, respectively) gametocytes, but the IC_50_s of R539T gametocytes were similar (1.9 and 2.2 µM, respectively) regardless of the duration of exposure. C580Y gametocytes displayed high variability in EI between independent replicates, as seen by the 95% confidence intervals (Fig. 1D). Despite this variability, highly significant differences could be observed using two isolates per K13 propeller group (Table 1), with a minimum of two replicates per isolate. Importantly, gametocytes from K13 propeller mutant isolates formed male gametes after exposure to DHA concentrations much higher than the physiologically relevant dose (700 nM) (6), suggesting that these could have a fitness advantage over WT gametocytes that enables them to persist in peripheral blood and remain functionally viable despite artemisinin treatment. Phenotyping of additional isolates in the EIA and determining whether K13 propeller mutant gametocytes are better able to form oocysts and sporozoites in the mosquito will further complement these observations and hypotheses.

To our knowledge, this is the first study demonstrating the effect of DHA on gametocytes produced *in vitro* from culture-adapted K13 propeller mutant Cambodian isolates. We have successfully used EIA to show that gametocytes from K13 propeller mutant slow-clearing isolates were more resistant to DHA than gametocytes from WT fast-clearing isolates, thereby providing a reliable assay with the potential to screen for antimalarials against mature gametocytes from drug-resistant clinical isolates.

## References

[1] WHO. 2015 Guidelines for the treatment of malaria, 3rd ed World Health Organization Press, Geneva, Switzerland.

[2] Noedl H, Se Y, Schaecher K, Smith BL, Socheat D, Fukuda MM 2008 Evidence of artemisinin-resistant malaria in western Cambodia. N Engl J Med 359:2619–2620. doi:10.1056/NEJMc0805011. 19064625

[3] Dondorp AM, Nosten F, Yi P, Das D, Phyo AP, Tarning J, Lwin KM, Ariey F, Hanpithakpong W, Lee SJ, Ringwald P, Silamut K, Imwong M, Chotivanich K, Lim P, Herdman T, An SS, Yeung S, Singhasivanon P, Day NP, Lindegardh N, Socheat D, White NJ 2009 Artemisinin resistance in Plasmodium falciparum malaria. N Engl J Med 361:455–467. doi:10.1056/NEJMoa0808859. 19641202 PMC3495232

[4] Ashley EA, Dhorda M, Fairhurst RM, Amaratunga C, Lim P, Suon S, Sreng S, Anderson JM, Mao S, Sam B, Sopha C, Chuor CM, Nguon C, Sovannaroth S, Pukrittayakamee S, Jittamala P, Chotivanich K, Chutasmit K, Suchatsoonthorn C, Runcharoen R, Hien TT, Thuy-Nhien NT, Thanh NV, Phu NH, Htut Y, Han K-T, Aye KH, Mokuolu OA, Olaosebikan RR, Folaranmi OO, Mayxay M, Khanthavong M, Hongvanthong B, Newton PN, Onyamboko MA, Fanello CI, Tshefu AK, Mishra N, Valecha N, Phyo AP, Nosten F, Yi P, Tripura R, Borrmann S, Bashraheil M, Peshu J, Faiz MA, Ghose A, Hossain MA, Samad R, 2014 Spread of artemisinin resistance in Plasmodium falciparum malaria. N Engl J Med 371:411–423. doi:10.1056/NEJMoa1314981. 25075834 PMC4143591

[5] Amaratunga C, Lim P, Suon S, Sreng S, Mao S, Sopha C, Sam B, Dek D, Try V, Amato R, Blessborn D, Song L, Tullo GS, Fay MP, Anderson JM, Tarning J, Fairhurst RM 2016 Dihydroartemisinin-piperaquine resistance in Plasmodium falciparum malaria in Cambodia: a multisite prospective cohort study. Lancet Infect Dis 16:357–365. doi:10.1016/S1473-3099(15)00487-9. 26774243 PMC4792715

[6] Witkowski B, Amaratunga C, Khim N, Sreng S, Chim P, Kim S, Lim P, Mao S, Sopha C, Sam B, Anderson JM, Duong S, Chuor CM, Taylor WR, Suon S, Mercereau-Puijalon O, Fairhurst RM, Menard D 2013 Novel phenotypic assays for the detection of artemisinin-resistant Plasmodium falciparum malaria in Cambodia: in-vitro and ex-vivo drug-response studies. Lancet Infect Dis 13:1043–1049. doi:10.1016/S1473-3099(13)70252-4. 24035558 PMC5015432

[7] Ariey F, Witkowski B, Amaratunga C, Beghain J, Langlois A-C, Khim N, Kim S, Duru V, Bouchier C, Ma L, Lim P, Leang R, Duong S, Sreng S, Suon S, Chuor CM, Bout DM, Ménard S, Rogers WO, Genton B, Fandeur T, Miotto O, Ringwald P, Le Bras J, Berry A, Barale J-C, Fairhurst RM, Benoit-Vical F, Mercereau-Puijalon O, Ménard D 2014 A molecular marker of artemisinin-resistant Plasmodium falciparum malaria. Nature 505:50–55. doi:10.1038/nature12876. 24352242 PMC5007947

[8] Amaratunga C, Witkowski B, Dek D, Try V, Khim N, Miotto O, Menard D, Fairhurst RM 2014 Plasmodium falciparum founder populations in western Cambodia have reduced artemisinin sensitivity in vitro. Antimicrob Agents Chemother 58:4935–4937. doi:10.1128/AAC.03055-14. 24867977 PMC4136061

[9] Straimer J, Gnädig NF, Witkowski B, Amaratunga C, Duru V, Ramadani AP, Dacheux M, Khim N, Zhang L, Lam S, Gregory PD, Urnov FD, Mercereau-Puijalon O, Benoit-Vical F, Fairhurst RM, Ménard D, Fidock DA 2015 Drug resistance. K13-propeller mutations confer artemisinin resistance in Plasmodium falciparum clinical isolates. Science 347:428–431. doi:10.1126/science.1260867. 25502314 PMC4349400

[10] Adjalley SH, Johnston GL, Li T, Eastman RT, Ekland EH, Eappen AG, Richman A, Sim BK, Lee MC, Hoffman SL, Fidock DA 2011 Quantitative assessment of Plasmodium falciparum sexual development reveals potent transmission-blocking activity by methylene blue. Proc Natl Acad Sci U S A 108:E1214–E1223. doi:10.1073/pnas.1112037108. 22042867 PMC3223476

[11] Price RN, Nosten F, Luxemburger C, ter Kuile FO, Paiphun L, Chongsuphajaisiddhi T, White NJ 1996 Effects of artemisinin derivatives on malaria transmissibility. Lancet 347:1654–1658. doi:10.1016/S0140-6736(96)91488-9. 8642959

[12] Targett G, Drakeley C, Jawara M, von Seidlein L, Coleman R, Deen J, Pinder M, Doherty T, Sutherland C, Walraven G, Milligan P 2001 Artesunate reduces but does not prevent posttreatment transmission of Plasmodium falciparum to Anopheles gambiae. J Infect Dis 183:1254–1259. doi:10.1086/319689. 11262208

[13] Chang H-H, Meibalan E, Zelin J, Daniels R, Eziefula AC, Meyer EC, Tadesse F, Grignard L, Joice RC, Drakeley C, Wirth DF, Volkman SK, Buckee C, Bousema T, Marti M 2016 Persistence of Plasmodium falciparum parasitemia after artemisinin combination therapy: evidence from a randomized trial in Uganda. Sci Rep 6:26330. doi:10.1038/srep26330. 27197604 PMC4873826

[14] WWARN Gametocyte Study Group. 2016 Gametocyte carriage in uncomplicated Plasmodium falciparum malaria following treatment with artemisinin combination therapy: a systematic review and meta-analysis of individual patient data. BMC Med 14:79. doi:10.1186/s12916-016-0621-7. 27221542 PMC4879753

[15] St. Laurent B, Miller B, Burton TA, Amaratunga C, Men S, Sovannaroth S, Fay MP, Miotto O, Gwadz RW, Anderson JM, Fairhurst RM 2015 Artemisinin-resistant Plasmodium falciparum clinical isolates can infect diverse mosquito vectors of Southeast Asia and Africa. Nat Commun 6:8614. doi:10.1038/ncomms9614. 26485448 PMC4616032

[16] Colmenarejo G, Lozano S, Gonzalez-Cortes C, Calvo D, Sanchez-Garcia J, Matilla JP, Leroy D, Rodrigues J 2018 Predicting transmission blocking potential of anti-malarial compounds in the mosquito feeding assay using Plasmodium falciparum male gamete inhibition assay. Sci Rep 8:7764. doi:10.1038/s41598-018-26125-w. 29773818 PMC5958111

[17] Amaratunga C, Sreng S, Suon S, Phelps ES, Stepniewska K, Lim P, Zhou C, Mao S, Anderson JM, Lindegardh N, Jiang H, Song J, Su XZ, White NJ, Dondorp AM, Anderson TJ, Fay MP, Mu J, Duong S, Fairhurst RM 2012 Artemisinin-resistant Plasmodium falciparum in Pursat province, western Cambodia: a parasite clearance rate study. Lancet Infect Dis 12:851–858. doi:10.1016/S1473-3099(12)70181-0. 22940027 PMC3786328

[18] Ruecker A, Mathias DK, Straschil U, Churcher TS, Dinglasan RR, Leroy D, Sinden RE, Delves MJ 2014 A male and female gametocyte functional viability assay to identify biologically relevant malaria transmission-blocking drugs. Antimicrob Agents Chemother 58:7292–7302. doi:10.1128/AAC.03666-14. 25267664 PMC4249523

[19] GraphPad. 2018 GraphPad Prism version 6.07 for Windows. GraphPad Software.

